# Podocyte Mitosis – A Catastrophe

**DOI:** 10.2174/1566524011307010013

**Published:** 2013-01

**Authors:** L Lasagni, E Lazzeri, S.J Shankland, H.-J Anders, P Romagnani

**Affiliations:** 1 Excellence Centre for Research, Transfer and High Education for the Development of DE NOVO Therapies (DENOTHE), University of Florence, Italy; 2 Division of Nephrology, Department of Medicine, University of Washington, Seattle, USA; 3 Medizinische Klinik und Poliklinik IV, Klinikum der Universität München, Munich, Germany; 4 Pediatric Nephrology Unit, Meyer Children’s Hospital, Florence, Italy

**Keywords:** Actin cytoskeleton, foot process, glomerulosclerosis, mitotic catastrophe, podocyte, renal progenitor.

## Abstract

Podocyte loss plays a key role in the progression of glomerular disorders towards glomerulosclerosis and chronic kidney disease. Podocytes form unique cytoplasmic extensions, foot processes, which attach to the outer surface of the glomerular basement membrane and interdigitate with neighboring podocytes to form the slit diaphragm. Maintaining these sophisticated structural elements requires an intricate actin cytoskeleton. Genetic, mechanic, and immunologic or toxic forms of podocyte injury can cause podocyte loss, which causes glomerular filtration barrier dysfunction, leading to proteinuria. Cell migration and cell division are two processes that require a rearrangement of the actin cytoskeleton; this rearrangement would disrupt the podocyte foot processes, therefore, podocytes have a limited capacity to divide or migrate. Indeed, all cells need to rearrange their actin cytoskeleton to assemble a correct mitotic spindle and to complete mitosis. Podocytes, even when being forced to bypass cell cycle checkpoints to initiate DNA synthesis and chromosome segregation, cannot complete cytokinesis efficiently and thus usually generate aneuploid podocytes. Such aneuploid podocytes rapidly detach and die, a process referred to as mitotic catastrophe. Thus, detached or dead podocytes cannot be adequately replaced by the proliferation of adjacent podocytes. However, even glomerular disorders with severe podocyte injury can undergo regression and remission, suggesting alternative mechanisms to compensate for podocyte loss, such as podocyte hypertrophy or podocyte regeneration from resident renal progenitor cells. Together, mitosis of the terminally differentiated podocyte rather accelerates podocyte loss and therefore glomerulosclerosis. Finding ways to enhance podocyte regeneration from other sources remains a challenge goal to improve the treatment of chronic kidney disease in the future.

## INTRODUCTION

The glomerular filtration barrier is composed of three layers: a glomerular basement membrane (GBM) lined on the blood side by the fenestrated glomerular endothelium and on the urinary side by a specialized epithelium, the podocytes. Increasing evidence suggests that the podocyte is the culprit in the great majority of glomerular diseases because loss of podocytes beyond a certain threshold induces glomerulosclerosis [1-4]. For example, podocyte numbers are reduced in proportion to the severity of injury and the magnitude of proteinuria is related to the extent of reduced podocyte number. Reduced podocyte number is a predictor of progression in patients with diabetic nephropathy, IgA nephropathy, and focal segmental glomerulosclerosis (FSGS) [5-7]. Independently from the type of insult (genetic, mechanical, immunological, toxic), podocytes respond in an apparently stereotypic pattern. They retract their foot processes – cellular extensions that cover the surface of the glomerular capillary loops - (foot processes effacement), detach from the GBM, and/or die [8]. These events impair the glomerular filtration barrier, which is clinically detectable as proteinuria. Foot process effacement involves the rearrangement of the well-ordered long actin fiber bundles into a dense network of disorganized filaments. Thus maintaining the sophisticated podocyte actin cytoskeleton is necessary to maintain two elementary functions of the foot process, adherence to the GBM and forming slit diaphragms between podocytes. Functional proof for the concept of cytoskeleton structure being mandatory for podocyte integrity comes from genetic studies in humans and mice documenting that mutations in distinct cytoskeletal podocyte proteins result in podocyte loss and glomerulosclerosis. In this review, we summarize the evidence on how the podocyte`s cytoskeleton is critical to maintain its functional and structural integrity. We then discuss how cellular responses that help most other cells to recover from injury often paradoxically leads to podocyte loss. Many findings, indeed, support the concept that although podocyte mitosis can occur, it represents a stressful event that drives podocyte loss either by detachment, death or both, explaining why response to injury requires hypertrophy of surviving podocytes or *de novo* podocyte formation.

## THE PODOCYTE’S STRENGTH AND WEAKNESS: ITS ACTIN CYTOSKELETON

Podocytes are polarized epithelial cells with an abundantly rich actin cytoskeleton and an arborized appearance with multiple major, intermediate, and minor foot process [8]. Foot processes of one podocyte inter-digitate with those of its neighbors to form a specialized cell-cell junction, the slit diaphragm, a multiprotein complex similar to adherent junctions covering filtration slits [8]. Foot processes are characterized by a podosome-like cortical network of short, branched actin filaments and by the presence of highly ordered, parallel contractile actin filament bundles [8], which are thought to modulate the permeability of the filtration barrier through changes in foot process morphology. Interference with any of the component of the actin cytoskeleton results in foot process effacement reflected by the retraction or fusion of the foot process and loss of the normal interdigitating pattern and proteinuria [9]. Thus, actin is the common denominator in both podocyte function and dysfunction.

### The cytoskeleton as a strength:

The apical membrane of the foot process is located on the podocyte luminal side, facing the urinary space, and it is characterized by a negative charged surface, due to the presence of the membrane proteins podocalyxin, podoplanin, and podoendin [10]. **Podocalyxin** functions also as anti-adhesin responsible for keeping the filtration slits open; it is associated with the actin cytoskeleton through the adaptor proteins Na^+^/H^+^ Exchanger Regulatory Factor (NHERF)-1 and -2 [11] and ezrin [12]. Loss of glomerular foot process associates with uncoupling of podocalyxin from the actin cytoskeleton [11]. Another protein located on the apical cell membrane of foot process is the glomerular epithelial protein 1 (**GLEPP-1**), a receptor tyrosine phosphatase involved in regulation of the podocyte cytoskeletal protein probably through direct or indirect interactions with vimentin [13].

At the basal membrane of foot process, two different sets of cell matrix adhesion complexes, integrins and dystroglycans, attach the intracellular actin cytoskeleton to the GBM through specialized junctions, known as the focal adhesion complexes [8]. Integrins, the major being α3β1s, bind to their ligands in the GBM and link the GBM to the intracellular actin cytoskeleton through paxillin, talin, vinculin, **α-actinin-4** and filamin [8]. Besides their adhesion functions, integrins also participate in “outside-in” and “inside-out” signaling. They regulate the actin cytoskeleton dynamics in response to extracellular stimuli by signaling through integrin-linked kinase (ILK) and the actin-related protein-2/3 (Arp2/3) complex [14]. The GBM is connected with the podocyte actin cytoskeleton also through α- and β-dystroglycan complex and utrophin [9].

The slit diaphragm proteins are connected to actin through a variety of adaptor and effector proteins. **Nephrin**, a member of the Ig-superfamily (Ig-SF) of transmembrane cell adhesion molecules, is one of the major structural components of the slit diaphragm [15]. The cytoplasmic tail of nephrin, like other transmembrane Ig-SF members, is anchored to the actin cytoskeleton of podocyte by means of CD2-associated protein, **CD2AP**, a cytoplasmic adaptor protein which interacts with actin [16] and the actin-binding proteins cortactin [17], capping protein Z (CapZ) [18], and the actin polymerization complex Arp2/3 [17]. CD2AP and nephrin interact with the p85 regulatory subunit of phosphoinositide 3-OH kinase (PI3K), recruit PI3K to the plasma membrane, and stimulate PI3K-dependent AKT signaling in podocytes [19], thus participating in a common signaling pathway necessary to maintain crucial podocyte functions. The external, highly glycosylated, Ig-like domain of nephrin forms dimers across the slit diaphragm, producing pores with a predicted diameter slightly smaller than the radius of albumin [20] and regulates glomerular permeability [15]. **Podocin** is another component of the slit diaphragm and its COOH-terminal cytoplasmic domain interacts with CD2AP and nephrin [21]. Podocin may serve two closely related functions: the recruitment and/or stabilization of nephrin at the podocyte foot process, and the augmentation of nephrin signaling, perhaps by organizing specialized nephrin-containing microdomains [22]. In lipid microdomains, podocin clusters and regulates the transient receptor calcium channel (TRPC)-6 [23] and it has been suggested that this regulation gives the slit diaphragm the ability to act as a mechanosensor that enables the podocyte to remodel its cytoskeleton and contract its foot process in response to mechanical stimuli [24]. Finally, **TRPC-6** is a critical controller of actin cytoskeleton. TRCP-6 activation increases cytoplasmatic Ca^2+^ levels, and its overexpression leads to loss of actin stress fibers in cultured podocytes and to proteinuria in mice [25]. A schematic representation of a latero-basal portion of a podocyte is given in Fig. (**1**).

### The cytoskeleton as a weakness:

Our understanding of the role of the cytoskeleton in podocyte function derives from known causes of hereditary human glomerular diseases and from studies on genetically manipulated animals. Mutations in the genes encoding nephrin and podocin were the first to be identified causing inherited isolated nephrotic syndrome (NS) [26, 27], representing the major genetic cause of congenital and infantile NS, respectively. The indispensable role of **nephrin** (**NPHS1**) at the slit diaphragm was shown further by the development of *NPHS1*-null mice, which displayed massive proteinuria and died within 24 h of birth [28]. Ten different **podocin **(***NPHS2***) mutations, comprising nonsense, frameshift and missense mutations, segregate with autosomal-recessive steroid resistant NS [27]. *NPHS2*-null mice develop severe proteinuria during the antenatal period and die a few days after birth from renal failure [29]. Podocalyxin/NHERF2/ezrin/actin interactions are disrupted in pathologic conditions associated with changes in podocyte foot process, providing evidence of its relevance *in vivo *[11]. Furthermore, podocytes of podocalyxin-null mice exhibit profound defects in kidney development and die within 24 hours of birth with anuric renal failure [30].

Mutations in the gene encoding α-actinin-4 (***ACTN4***) cause a late-onset autosomal dominant form of FSGS [31]. Mice with podocyte-specific expression of a high-affinity variant of *α*-actinin-4 [32] or genetic deletion of *α*-actinin-4 [33] or expressing *ACTN4 *gene harboring a disease-associated mutation are characterized by proteinuria with globally disrupted podocyte morphology in old mice [33]. Finally, *CD2AP*-null mice died at 6–7 weeks of age from renal failure [34] and mutations in the *CD2AP* gene leading to haplo-insufficiency have been identified in several families with FSGS [35]. Interestingly, combinations of *CD2AP* heterozygosity and heterozygosity of either *synaptopodin* or *Fyn* proto-oncogene resulted in spontaneous proteinuria and in FSGS-like glomerular damage. This suggests that combined mutations in two or more podocyte genes may be a common etiology for glomerular disease [36]. More recently, mutations in several other genes have been reported as causes of FSGS. Missense mutation in ***TRPC-6*** [37], which mediates hyperinflux of calcium in podocytes and constitutive activation of the calcineurin-NFAT pathway [38], causes familial FSGS. Alterations of the actin assembly Inverted Formin 2 **(*INF2*)** [39], which encodes a member of the formin family of actin-regulating proteins have been described in autosomal dominant FSGS. Moreover, mutation in ***Arhgap24*** [40], a RhoA-activated Rac1 GTPase-activating protein that controls actin polymerization, was associated with FSGS. Recently, mutations in myosin 1E (***MYO1E*)** an actin-dependent molecular motor, are associated with childhood-onset, glucocorticoid-resistant FSGS [41]. Finally, ***GLEPP1*-**null mice do not exhibit proteinuria, but podocytes display morphological changes, such as broadening of the foot process [13]. The slit diaphragm interactome is still growing, and the biological significance of individual molecules and interactions still needs clarification by future research. However, all these results underline the pivotal role of the actin cytoskeleton in the control of podocyte function, integrity and survival, as well as the critical role of actin disruption in causing podocyte loss.

## THE PODOCYTE’S CATASTROPHE: LOST CELL CYCLE CONTROL

### The Post-Mitotic State:

The necessity to maintain its complex cytoskeleton architecture is a major explanation why podocytes have a limited capacity to divide. Cells cannot simultaneously use their actin cytoskeleton for maintaining a sophisticated ultrastructure and for forming the mitotic spindle. The same applies to cells that use their actin cytoskeleton for migration. Indeed, in adherent or migrating cells, the actin network is rapidly dismantled and rearranged to allow the cell to form the mitotic spindle and to enter mitosis. Therefore, mitotic cells acquire a rather rounded shape. At the end of mitosis, actin forms part of the contractile ring [42]. This would result in loss of adherence with GBM and with adjacent podocytes, events that are all incompatible with maintaining podocyte function. This is why acquisition of functional specialization in a cell type (or a state of terminal differentiation), such as podocytes, neurons, cardiomyocytes is coupled with the permanent exit from the cell cycle [43] and the arrest in a “postmitotic” state, the molecular bases of which are not completely clarified. Forced re-entry of terminally differentiated cells into the cell cycle is however possible, as demonstrated by a number of experimental manipulations, such as viral infections [44, 45]*,* overexpression of cyclin D1 and cyclin dependent kinase (CDK)4/6 [46], ectopic expression of the Notch intracellular domain [47], or of elongation factor 2 (E2F) [48], but the consequences are often dramatic. In some instance, terminally differentiated cells indefinitely arrest in the G2 phase, otherwise, when full division occurs, it generates aneuploid cells that do not survive for long. Thus, far from being permanently postmitotic, terminally differentiated cells must continuously hold their cell cycle in check. When cell cycle control fails, the price of failure is high, and the death of cells may be an inexorable consequence.

### Fate of Terminally Differentiated Cells:

All these events have been described in detail in neurons that share many similarities with podocytes because the function of neurons and podocytes both largely depends on their structural interactions with other cells [49]. Replicative DNA synthesis in terminally differentiated neurons followed by failures of cell cycle completion is the main principle of cycle theory of Alzheimer’s disease (AD) [50, 51]. Vulnerable neurons of the AD brain exhibit biomarkers of cell cycle progression and DNA replication suggesting a re-entry into the cell cycle [52, 53]. A number of laboratories have reported the re-expression of various cell cycle proteins in neurons from patients with AD: cyclins A [52], B [52], D [53], and E [52], as well as CDKs [51], Proliferating Cell Nuclear Antigen (PCNA) [51, 52], Ki67 [52] and cyclin-dependent kinase inhibitors (CKIs) of both the Ink (Inhibitors of Kinases) and Cip/Kip (CDK interacting protein/Kinase inhibitory protein) families [54, 55]. One study demonstrated increased levels of phosphorylated H3 (Ser 10), a key regulator in chromatin compaction during cell division, in neuronal cytoplasm in AD compared to controls, further supporting the hypothesis that neurons in AD are mitotically activated [56]. It is notable that the presence of phosphorylated H3 in the neuronal cytoplasm may not be unique to AD. Indeed, a previous study showed increased levels of histones in the neuronal cytoplasmic pool in Huntington's disease brain [57]. There are also reports of cell cycle protein re-expression in amyotrophic lateral sclerosis [58], ataxia telangiectasia [59], Parkinson's disease [60], stroke [61] and other neurodegenerative conditions [62]. In any case, the aberrant expression and localization of phosphorylated histone H3, together with other cell cycle deregulation, was followed by cell death. This type of cell death that is triggered by aberrant mitosis and executed either during mitosis or in the subsequent interphase has been referred as mitotic catastrophe [63, 64]. Roninson *et al*. [65] defined mitotic catastrophe as a type of cell death resulting from abnormal mitosis, usually ending in the formation of nuclear envelopes around individual clusters of missegregated chromosomes. The result is the formation of large cells with multiple micronuclei and decondensed chromatin. Cells undergoing mitotic catastrophe usually do not show DNA ladder formation [66] or DNA breaks detectable by terminal deoxynucelotidyl transferase-mediated deoxyuridine triphosphate nick-end labeling (TUNEL) staining [67], suggesting that this cell death is non-apoptotic. However, recent studies suggest that it is inappropriate to define mitotic catastrophe on the basis of morphological criteria alone and proposed to functionally redefine mitotic catastrophe as an apical mechanism that senses mitotic failure and responds to it by driving the cell to an irreversible fate, be it apoptosis, necrosis or senescence. So far, at least three different programs of mitotic catastrophe have been described. First, cells can die without exiting mitosis (mitotic death); second, mitotic catastrophe can trigger a lethal pathway that is not executed until cells reaching interphase of the next cell cycle, or third, the cell can exit mitosis and undergo senescence [64]. Of note, in the adult neurons, death by cell cycle is a very slow process that can take from months to years, and might require an additional stimulus to make the transition from cycle to death.

### Cell Cycle and Mitotic Catastrophe:

**Fig. (2):**
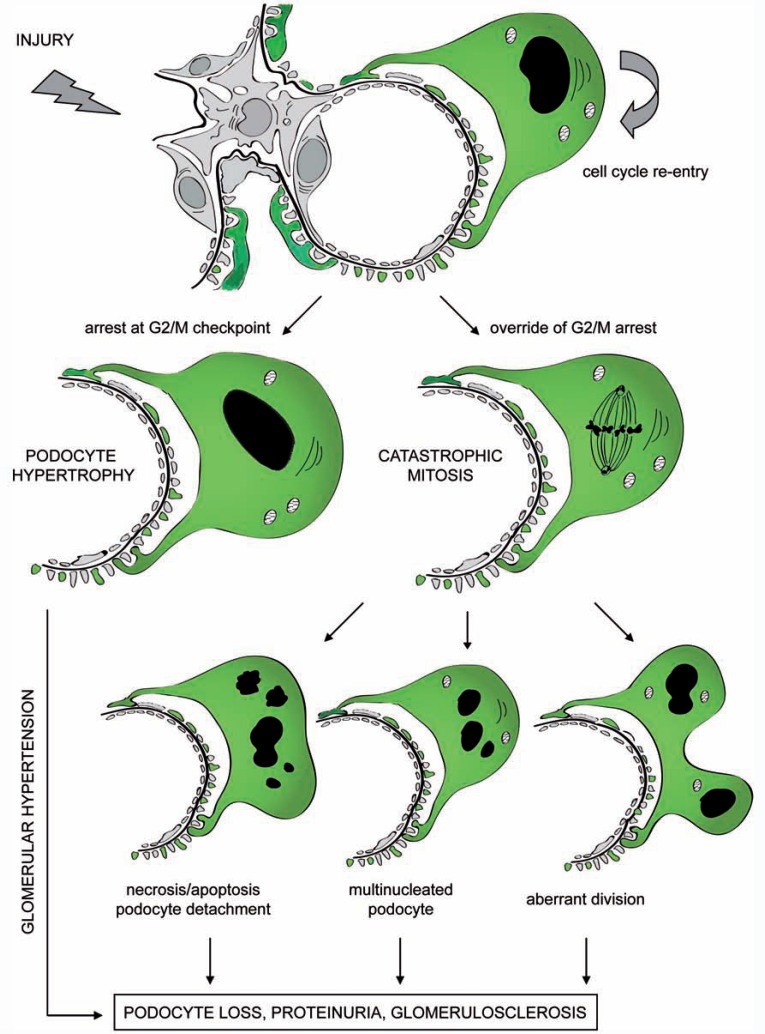
Pathological activation of podocyte cell cycle induces reentry into G1 and S-phases and initiation of DNA replication. Podocytes complete the DNA synthesis but cannot proceed through the M phase owing to the activation of mitotic catastrophe. Cells arrested in mitosis can have different fates: a) increase their size thus becoming hypertrophic; b) when division cannot be complete and cytokinesis fails, cells with gross nuclear alterations (multinucleation) are generated which quickly undergo “mitotic death” program; c) cells can exit mitosis containing a variable number of nuclei or micronuclei; these cells are viable because lethal pathway is not executed until cells reach interphase of the next cell cycle, but are unstable and detach from the GBM; in this case, cell death can occur in a delayed fashion even after years; d) when the aberrant division is productive aneuploidy cells form; most of these are unviable, owing to chromosomal rearrangements that result in progressive detachment to eliminate genomically unstable cells.

Several studies demonstrated the presence of an intrinsic barrier to replication associated with activation of cell cycle checkpoint also in podocytes. Re-expression of cell cycle proteins has been reported during glomerular disorders. *De novo* cyclin A staining was observed in podocytes of children collapsing glomerulopathy [68] and FSGS [69]; in cellular lesion of FSGS positive signals were also reported for cyclin D [70]; an altered expression of 27 and p21 was reported in patients with minimal change disease, collapsing glomerulopathy and FSGS [70]. Recently, strong up-regulation of the CKIs p21 and p27 was reported in podocytes during Heymann nephritis and diabetic ZDF-fa/fa rats [71, 72]. Moreover, involved glomerular tufts in crescentic glomerulonephritis strongly express CKIs [73]. These observations suggest that up-regulation of CKIs by podocytes is a general response to stress or injury. Interestingly, mechanical stretch of cultured podocytes also leads to increased CKIs, while stretch-induced growth arrest is absent in p21-/- podocytes [74], suggesting that podocytes up-regulate CKIs in order to maintain cell cycle quiescence and preserve normal physiological function. However, although podocytes resist entering the cell cycle, they can be forced at least as far as the mitosis phase under the pressure of sufficiently strong stimuli. We recently demonstrated that this forced entry in mitosis is a trigger for a catastrophic mitosis (Fig. **2**) because podocytes cannot assemble an efficient mitotic spindle due to poor expression of Aurora kinase B, which is essential for cytokinesis [75, 76]. Indeed, occurrence of abnormally mitotic podocytes and micro-multinucleation was demonstrated by electron microscopy in mice models of adriamycin nephropathy, a model of FSGS [75]. In adriamycin nephropathy, podocytes expressed histone H3, in the presence of nuclear abnormalities, which demonstrated the occurrence of mitotic catastrophe [75]. Interestingly, death through catastrophic mitosis was prevented by treating adriamycin nephropathy mice with inhibitors of the Notch pathway. These data suggest that Notch activation may represent an important driver of mitotic catastrophe in podocytes during glomerular disorders. Accordingly, Notch activation in differentiated podocytes induced aberrant mitoses, and the appearance of binucleated or micronucleated cells and cytoskeleton disruption. This was driven by a Notch-mediated downregulation of the cell cycle inhibitors p21 and p27, which forces progression toward mitosis of a cell that cannot assemble an efficient mitotic spindle, because it poorly expresses Aurora kinase B [75]. Interestingly, Notch expression is virtually absent in glomeruli of healthy adult kidneys, while several studies demonstrated strong Notch upregulation in podocytes of patients affected by several types of glomerular disorders characterized by podocyte death [75, 77, 78]. In addition, persistent activation of Notch in podocytes leads to podocyte loss and FSGS [75]. Although mitotic catastro-phe was not previously described as a mechanism of podocyte death, the presence of multinucleated podocytes has been non-specifically reported also in other experimental [79-86] as well human [87-92] glomerulopathies (Table **1**). For example, the presence of mitotic and multinucleated podocytes associated with heavy proteinuria and frequent glomerulosclerosis was reported as a consequence of forced re-entry into the cell cycle induced in rats by repeated injections of basic fibroblast growth factor (FGF) [79, 80]. Podocytes did not proliferate and lesions resembled classical synechiae. Interestingly, binucleate podocytes seemed to show foot process retraction with derangement of actin filaments [79, 80]. Forced cell cycle reentry of podocytes has been recently described as a consequence of conditional overexpression of telomerase reverse transcriptase (TERT) in transgenic mice [93]. TERT expression induced marked up regulation of Wnt signaling and disrupted glomerular structure, resulting in a collapsing glomerulopathy resembling those in human disease [93]. In addition, human renal biopsies reveal features of podocyte mitosis without cytokinesis, in glomerular disorders characterized by podocyte loss and proteinuria [personal communication]. Moreover, Hara *et al*. demonstrated the presence of many binucleate podocytes in the urine of patients affected by FSGS [94, 95] and lupus nephritis [95], suggesting that podocytes carrying nuclear abnormalities generated during an abnormal cytokinesis are more susceptible to detachment and loss.

In conclusion, the presence of cell cycle proteins, multinucleated podocytes or even DNA replication that are observed in podocytopathies on its own are not an evidence of local podocyte regeneration. These events may rather represent a process that ultimately paradoxically contributes to further podocyte loss leading to glomerulosclerosis, e.g. collapsing glomerulopathy. Some authors suggested that podocyte loss may involve podocyte apoptosis, but apoptotic podocytes remains rarely observed, even though mitotic catastrophe may involve subsequent apoptosis as one of several mechanisms how cells die upon aberrant mitosis.

## A LIFEBOAT FOR THE GLOMERULUS: THE RENAL PROGENITOR CELLS

### Podocyte Hypertrophy:

How does the podocyte layer compensate or adapt to podocyte loss? One response is that residual podocytes undergo an increase in cell size, i.e. hypertrophy. Podocyte hypertrophy is initially adaptive. This is an attempt by the cell that is relatively incapable of proliferating, to cover the underlying GBM in denuded areas where neighboring cells have detached or died. However, with time, podocyte hypertrophy becomes maladaptive. Indeed, loss of podocytes and segmental sclerosis lead to decreased ultrafiltration capacity and single nephron glomerular filtration rate (GFR) [96]. The compensatory mechanism that occurs to maintain a correct GFR in the hypertrophic glomerulus is glomerular hyperfiltration. However, glomerular hypertrophy and hyperfiltration might be both a cause and consequence of renal injury, contributing to the progressive deterioration of kidney function [97, 98]. Recently, Fukuda *et al*. [99] provided a mechanistic explanation for the “hyperfiltration hypothesis” demonstrating that local activation of the renin-angiotensin system within the glomerular tuft leads to an increase in angiotensin II concentration within the glomerulus. The first short-term effect of angiotensin is adaptive alterations in the podocyte cytoskeleton and increases in filtration pressures. However long-term local angiotensin effects contribute to an ongoing loss of podocytes by different mechanisms. Thus, pharmacological angiotensin II blockade can avoid inexorable progression to renal failure preventing podocyte depletion.

### Podocyte Regeneration:

**Fig. (3):**
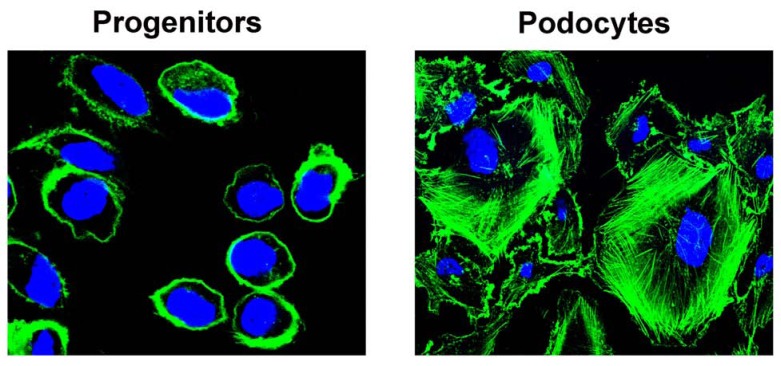
Phalloidin staining of F-actin showing the simple cytoskeleton of renal progenitors (left) in comparison to highly organized actin fibers present within podocyte cytoplasm (right).

Several studies also suggest that prolonged treatment with angiotensin converting enzyme inhibitors increases podocyte number in the absence of podocyte proliferation. These events are accompanied by and likely underlie the regression of glomerulosclerosis [100-102]*.* Regression of renal disease with remodeling of glomerular architecture has been reported in experimental animal models, and clinically in patients with different types of glomerular disorders who have received prolonged treatment with angiotensin converting enzyme inhibitors [102-104]. These results cannot simply be explained through the protective effects of angiotensin converting enzyme inhibitors on podocyte loss, but rather suggest that novel podocytes can potentially be generated. Recently, it was demonstrated that a population of progenitor cells in the parietal epithelium of the Bowman’s capsule of adult human kidney acts as a source of intra-renal progenitors for podocytes [105, 106]. These cells follow a phenotypical and functional hierarchy within the parietal epithelium of Bowman’s capsule. A population of renal progenitors committed towards becoming podocytes [106, 107] can be observed at the vascular pole. The existence of transitional cells, exhibiting a mixed phenotype between parietal epithelial cells and neo-podocytes in proximity of the vascular stalk of the glomerulus, has been reported also in other previous studies [108]. These podocyte progenitors are in direct continuity with fully differentiated podocytes and can proliferate and differentiate generating neo-podocytes in juvenile mice [109]. These cells have the potential to become podocytes without still displaying that complex cytoskeletal structure that constitutes such a major problem for an efficient mitotic division (Fig. **3**).

### Pro’s and Con’s of Glomerular Progenitors:

The discovery of progenitors brings with it a myriad of potential benefits and consequences. First, glomerular progenitors may make the prevention and treatment of glomerulosclerosis possible. However, this regenerative process can also sometimes be inadequate because of an inefficient or, in some situations, excessive, proliferative response [107, 110]. Converging evidence indicates the type of pathologic or clinic presentation, or even the outcome of glomerular disorders, may depend on the balance between injury of podocytes, and the regeneration provided by progenitors. Accordingly, very recent results suggest that a Notch-regulated balance between podocyte loss and regeneration provided by renal progenitors influences the outcome of glomerular injury in adriamycin nephropathy [75]. Indeed, in renal progenitors Notch activation stimulates entry into the S-phase of the cell cycle and cell division that is not hampered by the necessity to maintain a high complex cytoskeletal structure (Fig. **3**). Accordingly, inhibition of the Notch pathway in the regenerative phases of glomerular injury in mouse model of FSGS induced worsening of proteinuria and glomerulosclerosis [75]. Other still undiscovered factors, pathways and mechanisms may be implicated in the regulation of renal progenitor proliferation and differentiation. Recently, it has been demonstrated that β-catenin/Wnt signaling is required for proper cell fate decision of parietal epithelial cells. Indeed, in the glomeruli of the conditional *β*-catenin knockout mice, well differentiated podocytes replaced parietal epithelial cells in Bowman’s capsule [111]. Tracing nephrogenesis in embryonic conditional *β*-catenin knockout mice revealed that these “parietal podocytes” derived from precursor cells in the parietal layer of the S-shaped body by direct lineage switch. It has been also reported that increased Wnt expression was associated with loss of podocyte differentiation markers and expression of parietal cell type-specific markers, whereas deletion of Ctnnb1 increased the expression of podocyte markers [112]. However, it is possible that the renal effects of systemic manipulation of signaling pathways, such as Notch and Wnt, are highly context- and probably disease-dependent. Further studies will determine the role and contribution of specific signaling pathways in podocytes and progenitor physiology and the results of these studies may allow getting novel tools for the prevention and treatment of glomerulosclerosis.

## SUMMARY AND FUTURE DIRECTIONS

Converging evidence suggests that podocyte loss plays a key role in the progression towards glomerulosclerosis and chronic kidney disease, but the exact mechanisms causing the disease are not yet fully delineated. However, our understanding of the mechanisms leading to podocyte loss has increased significantly in the last decade. Several findings support the concept that podocytes possess an intrinsic barrier to proliferation due to their inability to assemble a correct mitotic spindle and perform a complete mitosis. Thus, a proliferative response of surviving podocytes often leads to detachment and death, now referred to as mitotic catastrophe, which instead of helping recovery from injury rather accelerates podocyte loss and glomerulosclerosis. Compensating for podocyte injury relies only on hypertrophy of surviving podocytes or *de novo* podocyte generation operated by renal progenitors. Finding ways to halt podocyte death or activating endogenous renal progenitors would contribute to reduce the burden of proteinuric renal diseases.

## Figures and Tables

**Fig. (1):**
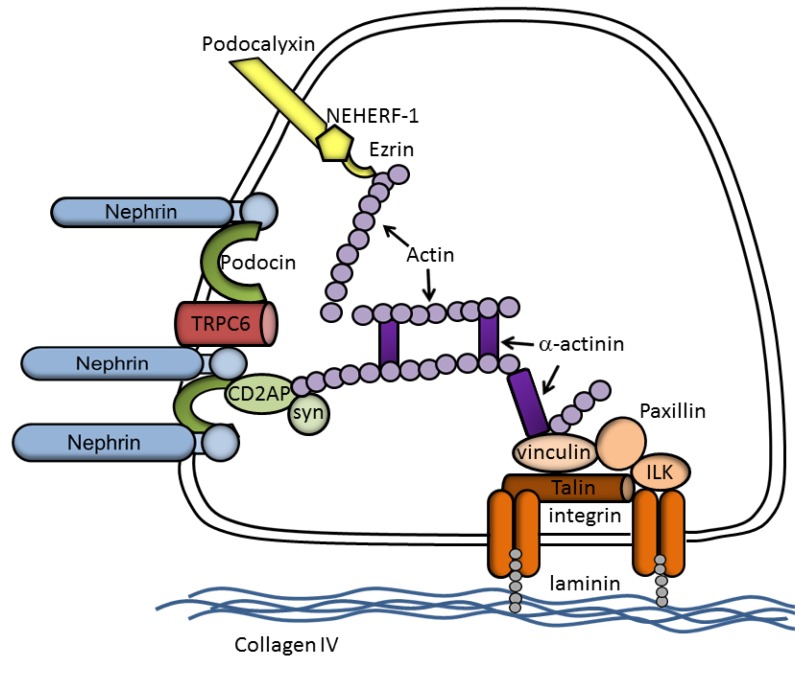
Schematic drawing of a latero-basal portion of a podocyte. In this greatly simplified graph, molecules are not drawn to a correct scale or shape.

**Table 1.** List of Experimental and Human Renal Pathologies with Alterations in Podocyte Nuclei

Pathologic Diagnosis	Light/Electron Microscopy	Reference
IgA, FSGS, Henoch-Schonlein nephritis, reflux nephropathy, lupus nephritis	Bi/polynucleated podocyte with extended cell cytoplasm.	[88]
PHN model of membranous nephropathy	Bi/multinucleated podocyte in treated animals	[79]
PHN model of membranous nephropathy	Polynucleated podocytes	[84]
PHN model of membranous nephropathy	Polynucleated podocytes	[83]
Collapsing glomerulopathy	Podocyte swelling, vacuolization, multinucleation	[87]
PAN rats given FGF2	Bi/polynucleated podocytes	[80]
FSGS with mitochondrial tRNALeu mutation	Bi/polynucleated podocytes	[91]
PAN, anti-Thy 1.1 nephritis, and 5/6-nephrectomy in rats	Bi/multinucleated podocytes in all disease models examined	[86]
Cystinosis	Multinucleated podocytes	[92]
Subtotal nephrectomy	Binucleated podocytes	[85]
PHN nephropathy PAN nephropathy Anti-GBM disease.	Bi/polinucleated podocytes	[82]

Abbreviations: PHN: passive Heymann nephritis; PAN: puromycin aminonucleoside.
